# Virtual Reality Portable Perimetry and Home Monitoring of Glaucoma: Retention and Compliance over a 2-year Period

**DOI:** 10.1016/j.xops.2024.100639

**Published:** 2024-10-29

**Authors:** Runjie B. Shi, Leo Y. Li-Han, Irfan N. Kherani, Graham E. Trope, Yvonne M. Buys, Willy Wong, Moshe Eizenman

**Affiliations:** 1 Temerty Faculty of Medicine, University of Toronto, Toronto, Ontario, Canada; 2 Institute of Biomedical Engineering, University of Toronto, Toronto, Ontario, Canada; 3 The Edward S. Rogers Sr. Department of Electrical & Computer Engineering, University of Toronto, Toronto, Ontario, Canada; 4 Department of Ophthalmology & Vision Sciences, University of Toronto, Toronto, Ontario, Canada

**Keywords:** Glaucoma, Visual field testing, Portable perimetry, Home monitoring, Progression

## Abstract

**Purpose:**

To evaluate long-term retention, compliance, and performance of glaucoma patients using a virtual reality portable perimeter to monitor visual fields (VFs) at home.

**Design:**

Prospective, longitudinal, cohort study.

**Subjects:**

Twenty-five glaucoma patients with stable and reliable VFs (average age 67.4 years) were recruited at Toronto Western Hospital, Ontario, Canada.

**Methods:**

Participants were instructed to perform bilateral home VF tests fortnightly for 2 years using the Toronto Portable Perimeter (TPP). Based on empirical home monitoring data, simulation analyses were conducted to evaluate the progression detection performance of high-frequency TPP testing.

**Main Outcome Measures:**

Retention rates were calculated as the percentage of participants who performed ≥1 home VF test. Compliance rates measured the percentage of participants adhering to the recommended test frequency of every 2-month period. Visual field indices, test reliability, intertest variability, and the precision of estimating progression rate with TPP were compared to those with the Humphrey Field Analyzer (HFA). After 6 months, participants completed a questionnaire to evaluate their experiences and preferences. The years required to detect progression were also compared between HFA and TPP tests.

**Results:**

Eighteen of the 25 participants (72%) completed ≥1 unsupervised VF test at home, with an average test frequency of 1.6 tests/month. Compliance decreased as the monitoring duration progressed, dropping from 83% (initial 2 months) to 11% (final 2 months). Unfamiliarity with technology and time constraints were identified as the main barriers to regular testing. Visual field indices of TPP home tests were strongly correlated with clinical results (*r* > 0.900). Home testing significantly reduced intertest variability (*P* < 0.001) and improved the precision of progression rate estimates (*P* < 0.010). Participants overwhelmingly preferred home testing over clinic VF follow-ups (*P* < 0.001). Simulations showed that TPP tests can significantly shorten the time to detect progression for different progression rates compared with clinical VF follow-up, even with compromised compliance.

**Conclusions:**

Despite the small sample size, our study demonstrated that glaucoma patients could reliably perform VF tests at home over a 2-year period. However, issues with retention rate and compliance with long-term VF monitoring were observed in some participants. Nevertheless, high-quality VF data from home tests can provide supplementary information to improve the timely detection of VF progression.

**Financial Disclosure(s):**

The author(s) have no proprietary or commercial interest in any materials discussed in this article.

Glaucoma, characterized by progressive and irreversible vision loss, is a leading cause of blindness globally.^1^ Visual field (VF) testing, or perimetry, is a standard clinical test of visual function in glaucoma patients. While glaucoma typically entails slow vision loss, certain patients may experience rapid deterioration.^2^ As such, continuous monitoring and timely detection of VF progression are essential for optimal glaucoma management.

To establish early detection, one strategy involves increasing the VF test frequency. Chauhan et al^3^ used simulations to demonstrate that conducting VF tests 3 times per year significantly reduced the time required to detect rapid progression compared with annual testing (decreased time to detection with 80% sensitivity from 6 to 2 years). Another simulation study by Anderson et al^4^ reported that fortnightly VF testing shortened the time to detect 80% of rapid VF progression in <2 years. Unfortunately, such high-frequency VF monitoring is impractical in a clinical setting due to limited medical resources and the additional cost to both patients and health care systems through increased clinical visits.

Recent advances with portable perimetry consistently show that VF tests performed with portable perimeters are comparable to those obtained with standard clinical devices,^5^, ^6^, ^7^, ^8^, ^9^, ^10^, ^11^ such as the Humphrey Field Analyzer (HFA; Carl Zeiss Meditec). Therefore, a number of studies have been undertaken to validate the feasibility of high-frequency home VF monitoring with portable perimeters. Jones et al^12^ conducted a monthly pilot study on 20 glaucoma patients at home over a 6-month period. Prea et al^13^^,^^14^ enrolled larger cohorts into 2 weekly home monitoring studies, each lasting no longer than 1 year. While these studies reported reasonably high patient compliance (69%–95%), the reliability of home VF tests was a concern, with around 40% of the home VF tests not meeting the reliability standard.^12^, ^13^, ^14^

The VF home monitoring studies mentioned previously used tablet-based perimeters. Tablet systems are susceptible to limitations, including variations in ambient lighting as well as fixation issues involving maintenance of a stable head-to-tablet distance, both of which impact test reliability.^12^, ^13^, ^14^ Moreover, a follow-up period of <12 months may be insufficient to evaluate a home VF monitoring setup for progression detection.^1^^,^^4^ Recently, virtual reality (VR) perimeters have gained prominence in VF testing for glaucoma patients,^5^^,^^6^^,^^8^^,^^9^ as head-mounted systems can overcome some of the limitations seen with tablet-based testing.

The primary objective of this study was to investigate the performance of a VR-based portable perimeter as a method to perform long-term home VF monitoring in glaucoma patients. Participants were asked to conduct VF tests twice a month at home using the Toronto Portable Perimeter (TPP)^5^^,^^6^ over a 2-year period. The TPP is a low-cost VR portable perimeter that has undergone extensive testing in glaucoma and optometry clinics and for visual screening outside clinics.^5^^,^^6^ Comparative analyses were made between the results of home vs. clinic VF tests. Participants were interviewed to understand their preferences as well as the reasons for their compliance level over the 2-year study duration. Furthermore, simulation studies were conducted based on the empirical data obtained from home monitoring to investigate the efficacy of VF progression detection.

## Methods

### Participants

Twenty-five glaucoma patients with known glaucomatous VF defects in 1 or both eyes were recruited from the Glaucoma Clinic at the Toronto Western Hospital, Ontario, Canada. Glaucoma diagnoses included normal tension glaucoma (N = 12), primary open-angle glaucoma (N = 6), primary angle-closure glaucoma (N = 3), pseudoexfoliation glaucoma (N = 2), and pigmentary glaucoma (N = 2). The inclusion criteria mandated no scheduled ocular surgery (including laser trabeculoplasty). Participants were required to have had ≥2 reliable VF tests performed on each eye utilizing the HFA 24-2 SITA standard algorithm, where the reliability criteria were defined as false-positive rate <20%, false-negative rate <20%, and fixation losses <33%. Patients with corrected visual acuity worse than 20/40 (logarithm of the minimum angle of resolution = 0.3) in either eye were excluded from the study. Additionally, patients who could not understand English instructions were also excluded.

Written consent was obtained from all participants before testing. This study received approval from the Research Ethics Board of the University Health Network in Toronto, Ontario, Canada, and adhered to the tenets of the Declaration of Helsinki.

### TPP

**Figure 1:**
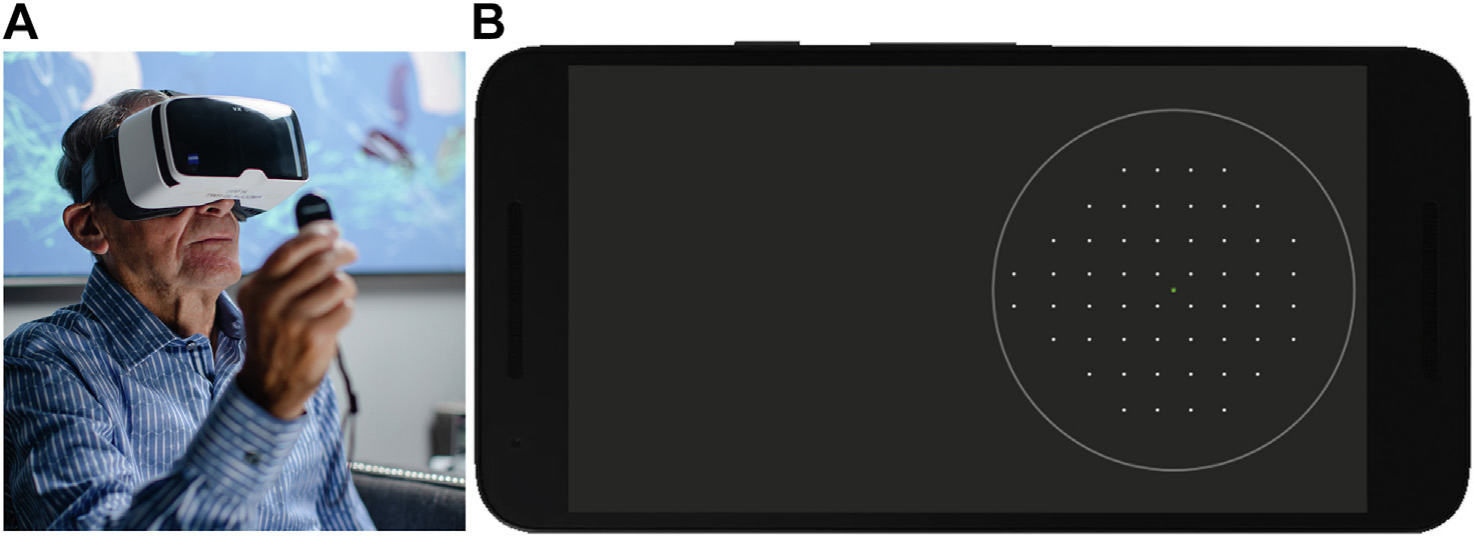
**A,** A glaucoma patient undergoing visual field testing using the Toronto Portable Perimeter (TPP) at home. **B,** Illustration of the standard 24-2 test pattern for the right eye on the smartphone’s screen of the TPP system.

The TPP consists of a personal smartphone, VR headset, wireless handheld clicker, and software to control and facilitate VF testing (Fig 1). During VF testing with the TPP, visual stimuli are presented on the smartphone’s screen and viewed through the optics of the VR headset, offering a field of view of ±30 degrees. Users maintain fixation on a central point displayed on the screen and press the handheld clicker whenever a stimulus is perceived. Throughout the test, the TPP system provides real-time audio feedback to alert users when they are not fixating on the central target (fixation loss) or when there are false-positive or false-negative responses.

Upon completion of each test, patient responses are processed by the TPP’s software and transmitted to a secure server for further data analysis and storage. Test results are presented in a report format consistent with the Single Field Analysis report generated by the HFA. The TPP test report comprises VF global indices, reliability indices, and test duration, as well as plots of differential light sensitivity thresholds, total deviations, and pattern deviations at each point tested. Visual field indices, including mean deviation (MD), pattern standard deviation (PSD), and VF index (VFI), are calculated based on the original papers^15^^,^^16^ and implementations.^17^ The report can be viewed by the users or clinicians locally on TPP’s smartphone and by remote access through the TPP data analysis platform.

### VF Home Monitoring Procedures

Each participant received a 20-minute tutorial on how to set up and use the TPP. After the tutorial, participants performed bilateral VF tests (24-2 TPP standard protocol) under the supervision of the research team. Subsequently, each participant was provided with a TPP device as well as printed instructions for VF home monitoring. Participants were instructed to complete 2 bilateral tests per month using the 24-2 TPP standard protocol for a duration of 2 years. The recommended frequency of testing was chosen based on the improved detectability of rapid progression as demonstrated in a simulation study.^4^

During the first 6-month period of testing, reminder emails were sent to encourage test adherence. Technical assistance was offered over the phone when patients encountered technical difficulties. At the first clinic visit following recruitment, participants were asked to complete a questionnaire designed to evaluate their experiences with home testing compared with clinical VF follow-up. This questionnaire comprised 14 questions, aiming to evaluate the usability of TPP, preference between TPP and HFA, and test convenience (e.g., the cost and time required for each clinical visit). These questions were developed based on a literature review, expert consultation, and validation. The questionnaire is included in Supplementary Method 1 (available at https://www.ophthalmologyscience.org).

Participants who discontinued testing before the end of the study period (including those who did not complete even a single test at home) were asked for the reasons for their decision. They were given several options to choose from, including (1) technological challenges, (2) time constraints (too busy to follow the test regimen), (3) health-related concerns or deteriorating health conditions, (4) demotivation, and (5) other reasons.

Although clinicians had access to the home visual field tests, they were not asked to review the results, as one of the main objectives of this research was to validate the feasibility of home monitoring. Instead, clinicians were provided with a 1-page report summarizing the patient's test results in a format similar to the progression analysis report at each clinical follow-up visit.

### Evaluation and Data Analysis

The retention rate was defined as the percentage of participants who successfully completed ≥1 TPP test at home. Compliance during each 2-month and 6-month period of the study was measured as the percentage of participants adhering to the recommended test frequency in each period.

Visual field indices (MD, PSD, and VFI) on the TPP during home monitoring and on the HFA in the clinic were compared for both eyes. Pearson correlation coefficients were calculated to measure the association between the TPP and HFA test results. Bland-Altman analysis was used to study the mean differences and 95% limits of agreements (LoA) between the 2 tests. The Wilcoxon signed-rank test was used to examine the differences between test reliability indices (false-positive rate, false-negative rate, and fixation losses) of the TPP and HFA tests. Furthermore, the percentage of TPP tests meeting the reliability criteria, referred to as the reliability rate, was compared to that of HFA tests.

As MD and VFI play essential roles in clinical VF progression analysis, we compared the intertest variability of MD and VFI from the HFA and the TPP tests. Given that all data were obtained from perimetrically stable patients, the intertest variability of MD and VFI was quantified by the root mean squared error between random pairs of VF tests for the same eye:

(1) $$RMSE=\sqrt{\frac{1}{M}\sum {\left(x_{i}-x_{j}\right)}^{2}},\forall \;i,j\in \left[1,n\right],i\neq j$$

where *x*_*i*_ and *x*_*j*_ denote 2 VF indices (MD or VFI) from the same eye using the same testing modality (HFA or TPP), *n* is the number of VF tests for this eye, and *M* is the total number of *x*_*i*_ and *x*_*j*_ pairs or combinations, i.e., *M*=*n*(*n*-1).

We also compared the repeatability coefficient (RC) of TPP home tests to that of the HFA clinic tests. The RC presents the value under which the absolute difference between repeated measurements is expected to fall with 95% probability,^18^^,^^19^ assuming there is no real change in patients’ VFs and the repeated measurements are independent. Repeatability coefficient is defined as:

(2) $$RC=1.96\sqrt{\frac{1}{N}\sum_{i=1}^{N}2Var\left(x_{i}\right)}$$

Here $x_{i}\in R^{k}$ represents k repeated measurements from the *i*-th subject, *Var*(**x**_*i*_) is the sample variance of **x**_*i*_, and *N* is the number of subjects. A lower RC represents better repeatability in the measurements.

Additionally, we investigated how high-frequency TPP tests at home affected the precision of estimating VF progression rates. In this investigation, the precision of estimating VF progression rate was quantified by the standard error (SE) of the linear regression slopes for MD and VFI. Note that the linear regression slope is the clinical standard measurement for VF progression rate. The standard error of a linear regression slope (*SE*_*β*_) can be expressed as:

(3) $$SE_{\beta }=\sqrt{\frac{1}{n-2}\frac{\sum_{i=1}^{n}{\left(y_{i}-{\hat{y}}_{i}\right)}^{2}}{\sum_{i=1}^{n}{\left(x_{i}-\bar{x}\right)}^{2}}}$$

where *y*_*i*_ and ${\hat{y}}_{i}$ are the *i*-th measured and predicted values of MD or VFI, respectively, *x*_*i*_ and $\bar{x}$ are the measured and mean values of the period of time from the first VF measurement, and *n* is the number of VF tests in the linear regression analysis. Only participants who have completed home VF testing over a period >1 year were included in this analysis.

The Wilcoxon signed-rank test was used to analyze statistical differences between the intertest variability, RC, and the precision of progression rates of MD and VFI, respectively. Moreover, we employed the Fisher exact test to compare the reliability rates between the TPP and the HFA tests and Chi-square tests to analyze questionnaire responses.

### Simulation of Progression Analysis with Home-Based VF Data

Given that participants remained perimetrically stable during the study period, we used simulations to evaluate the efficacy of high-frequency home tests for detecting VF progression. Early simulation studies relied on VF statistics generated in the clinical context using HFA. To better reflect the performance of home-based VF testing, we incorporated statistical and empirical features from our own home monitoring data, including baseline measurements, variability characteristics, and testing compliance.

Specifically, we utilized a data-driven simulation model previously introduced^20^^,^^21^ to generate longitudinal MD sequences with varying progression rates (0, −0.5, −1.0, −1.5, and −2.0 decibels [dB]/year) and testing frequencies (semiannual and fortnight). This approach differs from previous methods^20^^,^^21^ by incorporating the empirical MD test-retest variability and test compliance data from our study to simulate high-frequency home VF testing. For each selection of the progression rate and test frequency, we generated 10 000 MD sequences with the initial MD value randomly selected based on the data of TPP home tests. To simulate the worsening of compliance over time, we estimated time-varying compliance for all patients, constructing a pool of compliance rate time series. For each simulated MD sequence, a compliance rate time series was randomly selected from the pool. Based on this compliance series, (1 - compliance rate)% of tests were deleted to represent the compliance pattern of home VF testing.

The simulated MD sequences from TPP and HFA tests were then used to detect VF progression, defined as a statistically significant negative MD slope (*P* < 0.050).^4^ The detection sensitivity, specificity, and time required to detect 80% of progressions with simulated TPP home tests were compared with those obtained from simulated HFA tests. Further details on the simulation methodology can be found in Supplementary Method 2 (available at https://www.ophthalmologyscience.org).

## Results

### Demographics

Table 1 details the participants’ demographics. We interviewed >100 patients who met the inclusion criteria, of which 25 (12 females and 13 males) consented to this study. All participants had >2 years of VF testing experience and ≥2 reliable tests. The average age (± standard deviation) of the participants was 67.4 ± 8.1 years, ranging from 48 to 80 years. At the time of recruitment, the MD for the HFA tests averaged over all participant eyes was −5.2 ± 4.2 dB, ranging from −14.8 dB to 1.7 dB. All participants were confirmed to be perimetrically stable on HFA throughout the 2-year study period.

**Table 1:** Participants Demographics

	Count	Age (Mean [Range], Unit: Years)	Initial Mean Deviation∗ (Mean [Range], Unit: dB)		Diagnosis			
			OD (Right Eye)	OS (Left Eye)	POAG	NTG	PACG	Others†
Female	12	63.0	−3.5	−7.8	4	4	2	2
		[48.1, 72.0]	[–13.2, 0.4]	[−11.2, −5.4]				
Male	13	70.9	−4.3	−5.3	2	8	1	2
		[61.0, 80.3]	[–14.8, 1.7]	[–8.2, 0.5]				
All	25	67.4	−3.9	−6.4	6	12	3	4
		[48.1, 80.3]	[–14.8, 1.7]	[–11.2, 0.5]				

dB = decibels; NTG = normal tension glaucoma; PACG = primary angle-closure glaucoma; POAG = primary open-angle glaucoma.

∗ Initial mean deviation (MD) refers to the MD value obtained from the Humphrey Field Analyzer at the time of recruitment.

† Others including pseudoexfoliation glaucoma (N = 2) and pigmentary glaucoma (N = 2).

### Retention and Compliance

Of the 25 patients enrolled, 18 successfully carried out unsupervised VF tests at home using TPP, resulting in a retention rate of 72%. Eighteen retention patients achieved an average test frequency of 1.6 tests/month over the 2-year period, with results ranging from 0.2 to 4.3 tests/month. Eleven of the 18 retention participants (61%) performed VF testing at an average frequency >1 test/month, and 6 participants (33%) achieved a test frequency >2 tests/month. Thirteen of these 18 patients (72%) completed regular home VF tests for >1 year, with an average test frequency of 2.0 tests/month (range: 0.6–4.3 tests/month).

Compliance over different stages of the study showed a markedly decreasing trend. During the initial 6 months of home monitoring, all 18 participants actively participated in home VF testing, with 15 participants (83%) completing ≥1 test per month and 12 (67%) achieving the recommended frequency of 2 tests/month. By comparison, the number of active participants dropped to 12 (67%) during the final 6 months of the study, with 8 patients (44%) conducting VF tests monthly and only 2 participants (11%) adhering to the recommended fortnightly testing frequency. The 2 primary reasons given for discontinuing home testing were unfamiliarity with technology and time constraints.

**Figure 2:**
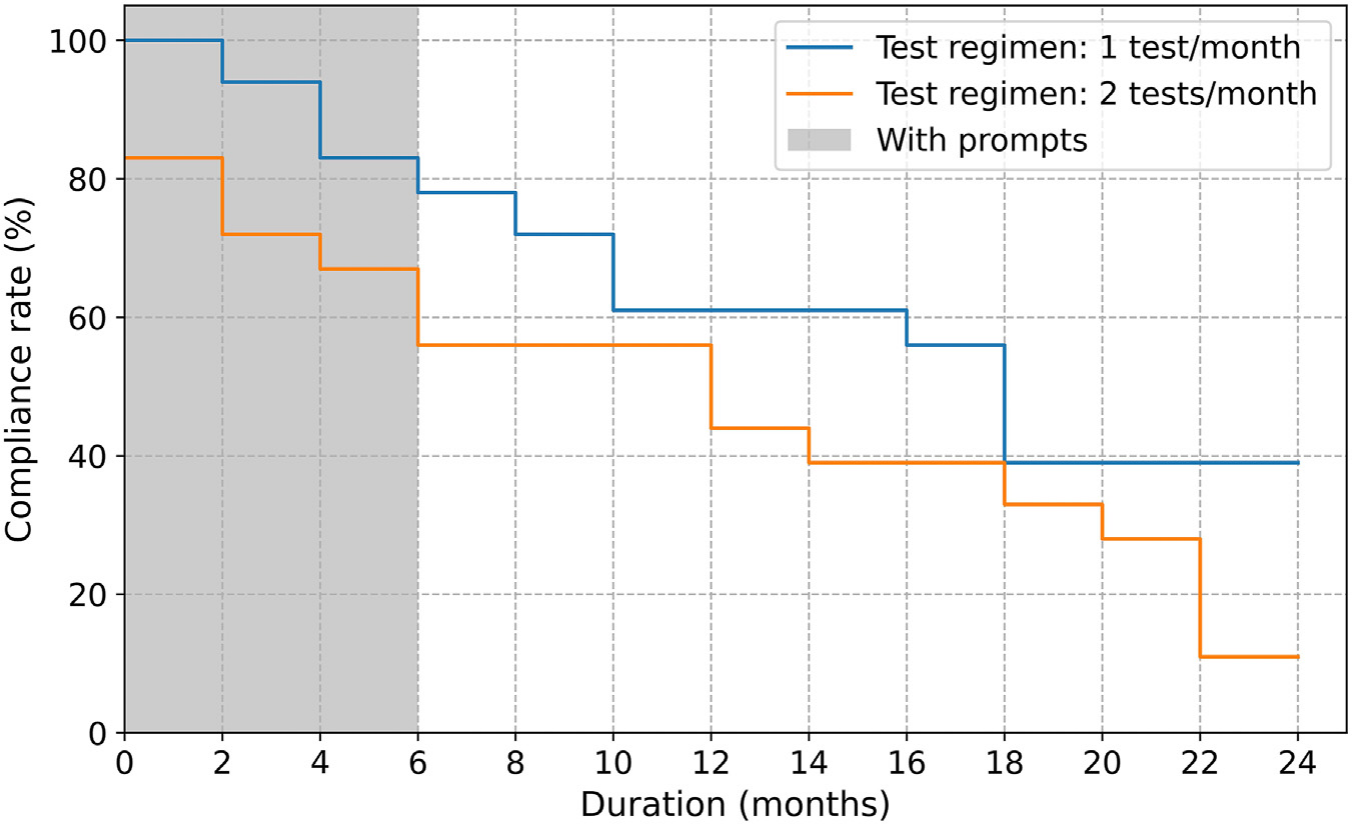
The relationship between the 2-month compliance rate and the duration of home monitoring with 2 different testing schedules (1 test/month and 2 tests/month). The shaded area in the first 6 months of the monitoring represents the period during which prompts were sent to all participants.

**Figure 3:**
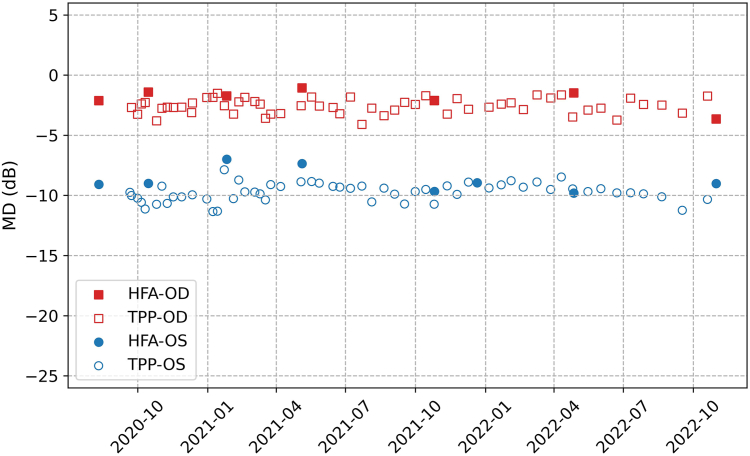
Mean deviation (MD) results from Humphrey Field Analyzer (HFA) and Toronto Portable Perimeter (TPP) for both eyes of 1 participant who have completed home visual field testing with the mean test frequency of 2.2 tests per month for >2 years. The squares and circles represent the MD for right (OD) and left (OS) eyes, respectively, while the filled and empty markers stand for the measurements from HFA and TPP.

Table 2 provides a breakdown of testing compliance during different 6-month intervals throughout the 2-year study. Figure 2 details the compliance rates for testing frequencies of 1 and 2 per month within each 2-month period. Figure 3 provides a quantitative illustration of MD measurements from VF tests conducted on 2 eyes of 1 participant. This particular patient engaged in VF home monitoring for a duration of 2 years, with a mean testing frequency of 2.2 tests per month.

**Table 2:** Testing Compliance in Different 6-Month Periods of the Visual Field Home Monitoring Study with the Toronto Portable Perimeter

Monitoring Period	Number of Active Subjects∗	Test Frequency (Mean [Range], Unit: Tests/Month)	Compliance Rate with 1 Test Per Month†	Compliance Rate with 2 Tests Per Month
0–6 months	18	2.4 [0.7, 4.4]	83%	67%
6–12 months	17	1.8 [0.3, 4.4]	67%	44%
12–18 months	13	2.0 [0.5, 4.4]	56%	33%
18–24 months	12	1.8 [0.3, 4.3]	44%	11%
0–12 months	18	2.0 [0.4, 4.3]	78%	56%
0–24 months	18	1.6 [0.2, 4.3]	61%	33%

∗ An active subject is defined as a participant who performed ≥1 home visual field test in the specified period.

† Compliance rates represent the percentage of participants (out of 18) conducting home visual field tests following the frequency of 1 or 2 tests/month.

### VF Global Indices

We analyzed 1434 TPP home tests and 184 HFA clinical tests from all 18 participants. As all patients were perimetrically stable throughout the study, we compared the average of TPP tests for each eye with those of HFA tests.

**Figure 4:**
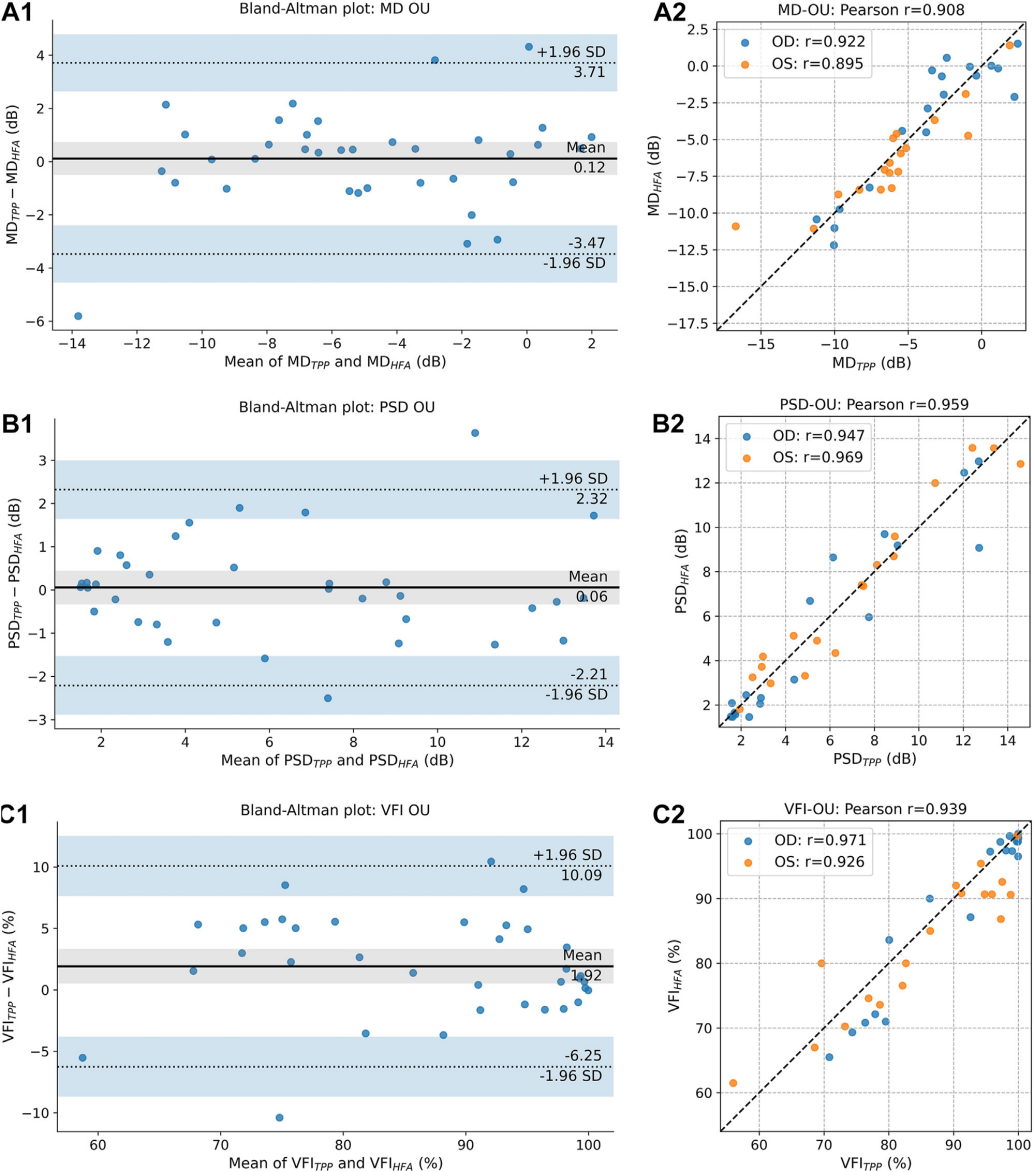
Bland-Altman and scatter plots of MD (**A**), PSD (**B**), and VFI (**C**) between visual field tests from the HFA and the TPP for 36 eyes of 18 glaucoma patients. dB = decibels; HFA = Humphrey Field Analyzer; MD = mean deviation; OD = right eye; OS = left eye; OU = both eyes; PSD = pattern standard deviation; SD = standard deviation; TPP = Toronto Portable Perimeter; VFI = visual field index.

Figure 4 illustrates the Bland-Altman and scatter plots for MD, PSD, and VFI for the HFA and TPP tests. Visual field indices from TPP and HFA tests exhibited strong associations (*P* < 0.001), with a Pearson correlation coefficient of 0.908, 0.959, and 0.939 for MD, PSD, and VFI, respectively. Bland-Altman analyses indicated no significant differences between TPP and HFA tests in MD and PSD. Specifically, the mean difference (TPP minus HFA) for MD was 0.12 dB (Wilcoxon *P* = 0.470), with 95% LoA ranging from −3.47 to 3.71 dB. Similarly, the mean difference for PSD was 0.06 dB (Wilcoxon *P* = 0.957), and the 95% LoA was −2.21 to 2.32 dB. The mean VFI of tests on the TPP was higher than that of tests on the HFA, with a mean difference of 1.92% (Wilcoxon *P* = 0.006) and 95% LoA from −6.25% to 10.09%. Furthermore, the point-wise sensitivity between TPP and HFA tests also demonstrated strong correlations, with the mean (standard deviation) Pearson correlation coefficient of 0.878 (0.081).

### Test Reliability

Both HFA and TPP tests demonstrated a high level of reliability. Based on the criteria used for the recruitment of patients, 83% of the TPP home tests (1191/1434) were categorized as reliable, while the reliability rate for HFA clinical tests was 86% (158/184, Fisher exact test *P* = 0.400). Toronto Portable Perimeter tests showed slightly higher mean false-positive and mean false-negative rates (4.7% and 5.0%, respectively) compared to the rates of HFA tests (3.7% and 3.1%, respectively). The mean fixation loss rate for TPP tests (9.6%) was lower than that of the HFA tests (12.3%). While a statistically significant difference was observed with the false-negative rates between TPP and HFA tests (5.0% vs. 3.1%, Wilcoxon *P* = 0.002), this has likely limited impact on result interpretation given that both values fall well below the study’s reliability criterion (20%).

### Intertest Variability, Repeatability, and Precision

**Figure 5:**
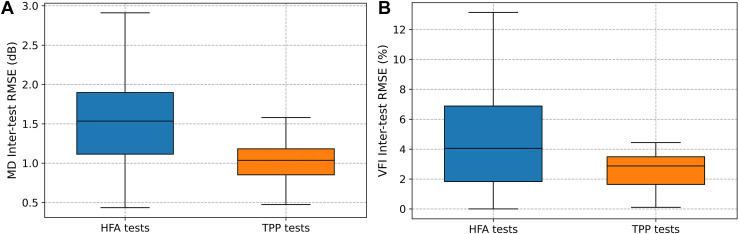
Boxplots of the intertest variability for MD (**A**) and VFI (**B**) of visual field tests from the HFA (blue) and the TPP (orange). dB = decibels; HFA = Humphrey Field Analyzer; MD = mean deviation; RMSE = root mean squared error; TPP = Toronto Portable Perimeter; VFI = visual field index.

To examine the repeatability of high-frequency VF testing, we compared the intertest variability of MD and VFI obtained from TPP home tests, as defined in Equation (1), with those from the clinical HFA tests. As shown in Figure 5, the intertest variability of MD measurements decreased from 1.67 ± 0.92 dB for HFA tests to 1.18 ± 0.65 dB for TPP tests (Wilcoxon *P* < 0.001). Similarly, the intertest variability of VFI for TPP tests (2.84 ± 2.00%) was lower than the 4.53 ± 3.30% for HFA tests (Wilcoxon *P* < 0.001). Furthermore, the RC of MD for TPP tests (3.0 dB) was lower than HFA tests (3.4 dB, Wilcoxon *P* = 0.227). The RC for TPP’s VFI (7.1%) outperformed that for the HFA results (9.9%, Wilcoxon *P* = 0.012).

**Figure 6:**
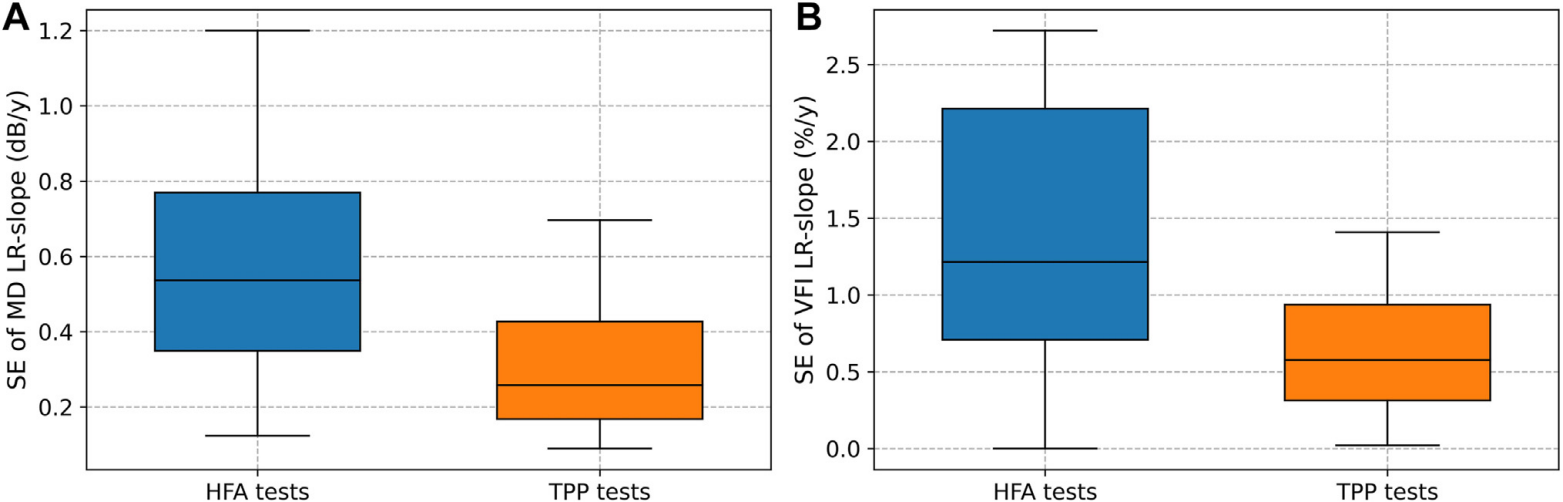
Boxplots of the SE (precision) of linear regression (LR) slopes for MD (**A**) and VFI (**B**) using data from the HFA (blue) and the TPP (orange). dB = decibels; HFA = Humphrey Field Analyzer; MD = mean deviation; SE = standard error; TPP = Toronto Portable Perimeter; VFI = visual field index.

Figure 6 illustrates the boxplots of the SE of linear regression slopes using data from the TPP and the HFA tests. As can be seen, high-frequency TPP tests resulted in a reduced SE of MD slope, decreasing from 0.59 ± 0.29 dB/year with HFA data to 0.38 ± 0.31 dB/year when using TPP tests (Wilcoxon *P* = 0.003). A similar improvement in the SE (i.e., precision) of VFI progression rate estimates can also be observed when the data of TPP tests was used for estimating the slope, with the SE of VFI slope decreasing from 1.47 ± 1.02%/year for the HFA tests to 0.96 ± 1.18%/year for the TPP tests (Wilcoxon *P* = 0.005).

### Questionnaire

Table 3 summarizes the results of seven multiple-choice questions completed by the 18 patients who participated in this study. All participants preferred home testing (88.9%, N = 16) or had no preference (11.1%, N = 2) vs. clinic testing (*χ*^2^ *P* < 0.001). Ten of the 18 participants (55.6%) indicated that TPP produced less anxiety during the VF testing compared with 2 (11.1%) who felt less anxiety with the HFA (*χ*^2^ *P* = 0.069). The majority of participants (94.4%, N = 17) stated that they would choose TPP as the testing tool if they were asked to perform multiple VF tests vs. using the HFA (N = 1, *χ*^2^ *P* < 0.001). Additionally, a higher percentage of participants found the voice-guided instructions from the TPP to be easier to understand (38.9%, N = 7) compared with 16.7% (N = 3) who preferred instructions from the HFA clinic technicians, while 8 other participants (44.4%) reported no difference between the 2 testing modalities.

**Table 3:** Summary of the Questionnaire Responses for Visual Field Home Monitoring with the TPP

Questions	Home	Clinic	No Preference	*P* Value∗
Where would you prefer to perform your visual field test if given a choice?	16 (88.9%)†	0 (0)	2 (11.1%)	<0.001

	TPP	HFA		

Which test would you prefer if you were to complete multiple visual field tests?	17 (94.4%)	1 (5.6%)	0 (0)	<0.001
Which test produced the least amount of anxiety?	10 (55.6%)	2 (11.1%)	6 (33.3%)	0.069
Which test instructions were easier to understand?	7 (38.9%)	3 (16.7%)	8 (44.4%)	0.311
Which test did you find easier to perform?	5 (27.8%)	3 (16.7%)	10 (55.6%)	0.114

	Yes		No	

Do you take time off from work to complete an in-hospital visual field test?	7 (38.9%)		11 (61.1%)	0.346
Do you require someone to accompany you to the hospital during follow-up visits?	3 (16.7%)		15 (83.3%)	0.004

HFA = Humphrey Field Analyzer; TPP = Toronto Portable Perimeter.

∗ Chi-square test.

† Data are presented in the form of count (percentage).

The average travel time spent by the 18 participants on transportation to attend VF follow-up visits was 3.5 ± 5.7 hours (round trip), ranging from 0.5 to 24 hours. The average cost for the VF follow-up visits, including transportation and accommodation (if applicable), was $50.3 ± 85.5 (range: $5 to $355, in Canadian dollars). In addition, 7 of 18 participants (39%) had to take time off from work to attend clinical VF tests, and 3 participants (17%) needed accompanying individuals for their clinical visits.

### Time to Detection

Table 4 summarizes the time required to detect 80% of simulated VF progression for clinic and home tests at different VF progression rates. Note that the simulated TPP tests followed the same variability and compliance as observed in real patients. With fortnight testing at home (using the TPP), it would take 3.4, 2.2, 1.8, and 1.4 years to achieve 80% sensitivity to detect a MD progression of −0.5 dB/year, −1 dB/year, −1.5 dB/year, and −2.0 dB/year, respectively. With standard semiannual HFA testing at the clinic, it would take 6.8, 4.3, 3.4, and 2.8 years, respectively, to reach the same detection power. The detection specificity was maintained at around 95% in all the simulations. Note that the RCs for the simulated HFA and TPP tests (3.3 dB and 2.9 dB, respectively) were approximately the same as those measures from the VF data of glaucoma patients in our study. The consistency of the RCs suggests that the simulated HFA and TPP data maintained similar variability characteristics as measured VF data from patients.

**Table 4:** Years Required to Detect 80% of the Progression in Simulated Longitudinal HFA and TPP Data with Different Underlying True Progression Rates in Mean Deviation

Data	Time (Years) to Detect 80% of the Progression			
	−0.5 dB/Year	−1.0 dB/Year	−1.5 dB/Year	−2.0 dB/Year
Clinic HFA tests∗	6.8	4.3	3.4	2.8
Home TPP tests†	3.4	2.2	1.8	1.4

dB = decibels; HFA = Humphrey Field Analyzer; TPP = Toronto Portable Perimeter.

∗ HFA tests were simulated with a testing interval of 6 months.

† Home TPP tests were simulated using a testing frequency of 2 tests/month, following the observed compliance pattern of home monitoring. The detection specificity maintained around 95% in all cases.

## Discussion

With rapid advances in portable perimetry technology, it is now possible to use home-based VF testing as a method for detecting and monitoring glaucoma. In this study, we reported that glaucoma patients can perform reliable VF tests at home using a VR-based portable perimeter (TPP) over a prolonged period of time. Although the majority of our subjects failed to achieve the ideal testing frequency of twice a month, participants who completed the study (72%) were able to maintain an average frequency of 1.6 tests/month over a 2-year period. Visual field tests at home demonstrated high reliability and strong correlations with tests conducted at the clinic. Furthermore, frequent home VF tests reduced the variability of differential light sensitivity measurements and therefore improved the precision by which VF progression rates could be estimated. To the best of our knowledge, this study is the longest exploration of self-administered home testing with VR portable perimetry to date, offering insights into the retention and compliance of long-term, high-frequency monitoring at home.

### Importance of VF Home Monitoring

The study period for most participants coincided with the onset of the coronavirus disease 2019 pandemic. This unprecedented situation highlights the pressing need for methods to remotely monitor and manage chronic conditions like glaucoma. The questionnaire responses underscored the acceptance of home-based VF testing among glaucoma patients. We report an overwhelming preference for home VF testing with TPP over clinical VF follow-up with HFA (>88% of the participants). More generally, home-based VF monitoring is in line with the increasing use of telehealth during and after the pandemic.^22^, ^23^, ^24^ In this context, our study can serve as a real test regarding the practicality and effectiveness of long-term VF home monitoring.

We found, however, certain challenges to adopting long-term home VF monitoring. Notably, our sample size was limited to 25 patients, with 28% of this group (7 patients) unable to complete even a single VF test at home. Also, despite regular prompting, the test frequency varied significantly among participants, with testing intervals ranging from less than a week to 6 months. This observation indicates that some patients are highly motivated to perform VF tests at home, while others lack the ability or motivation to conduct frequent VF tests over the long term. Therefore, high-frequency home monitoring may not be suitable for everyone, which may require special considerations. One possible solution is to adopt customized follow-up and reminder strategies for different patients or perhaps to integrate VF tests with other routine care like blood pressure monitoring, etc. Further details regarding testing compliance and challenges are discussed separately later.

### High-Frequency and Repeatable VF Testing for Progression Analysis

Among the different VF home monitoring studies conducted thus far,^12^, ^13^, ^14^ the current study has yielded the lowest RC for home-based VF tests. A lower RC indicates less variation in the results, i.e., a better outcome. Moreover, the RC for MD obtained with TPP at home was 12% lower than that of HFA tests conducted at the hospital. We believe the improved repeatability of TPP tests compared with HFA tests can be partially attributed to frequent testing, which provides more practical experiences with the same device in the same environment. By comparison, the relatively large interval between HFA tests (e.g., ≥6 months) may necessitate a relearning process for the patient in each follow-up visit, compromising the test results. Moreover, the more relaxed and quieter environment at home than in the clinic may also contribute to the improvement in repeatability.

In general, the VR headset, equipped with a strap and a light seal, provides a stable and uninterrupted testing environment at home, closely resembling conditions in a quiet exam room, further enhancing test repeatability. Additionally, VR-based testing eliminates the need for users to actively maintain a fixed distance from the screen needed by tablet perimeters. These advantages are also reflected in the higher testing reliability of VR-based perimetry compared with tablet-based VF tests. In our study, 83% of the TPP home tests were categorized as reliable. By comparison, the reliability rate for tablet-based VF tests reported in early VF home monitoring studies was significantly lower at around 60%.^13^^,^^14^

To demonstrate the importance of lower VF measurement variability for progression detection, our simulation analysis showed that with fortnight VF testing on the TPP, the sensitivity for detecting VF progression of −1 dB/year in 2 years was 0.75. That is, even with the reduced testing compliance observed in our study, >75% of rapid and catastrophic VF progressions (deteriorating worse than −1 dB/year) can likely be detected statistically within 2 years. In contrast, the 2-year detection sensitivity for a −1 dB/year progression rate was 0.64 when HFA tests were simulated on a fortnightly basis, using the same compliance as TPP home tests. These results suggest that the better repeatability of TPP tests at home relative to HFA tests in the clinic will likely lead to a marked improvement in detection sensitivity of VF progression, from 0.64 to 0.75—an increase of 17%. Finally, the simulated 2-year detection sensitivity can exceed 0.95 if all home VF tests are conducted consistently twice per month, i.e., 100% testing compliance.

### Testing Compliance and Challenges with VF Home Monitoring

Figure 2 shows the inverse relationship between the compliance and the duration of home monitoring found in our study. This trend persists irrespective of the target test frequency or the presence of prompts. The decrease in compliance can be associated with a reduction in engagement with the testing (see Table 2). We suggest that the reason for the decline may arise from the patient’s lack of understanding of the test results (as compared to blood pressure measurements, which are easier to interpret). As such, a robust and easy-to-interpret scale like “same/better/worse” may help patients better understand disease progression, appreciate the importance of regular VF testing, and alleviate the anxiety of uninterpretable testing results. However, determining the optimal way to present and interpret high-frequency home monitoring results remains a topic for future research. Additionally, it is important to note that the compliance rates observed in our study may also be overestimated due to possible selection bias, as we were only able to recruit patients willing to participate.

Seven participants withdrew from our study, and an additional 5 of the remaining 18 participants terminated testing within 1 year. In a subsequent interview, the 2 primary reasons for their withdrawal were (1) challenges with technology and (2) time constraints. Similar results have been reported with other VF home monitoring studies.^13^^,^^14^ Five participants who considered technical difficulties the biggest barrier were typically older participants, with a mean age of 72.5 years. On the other hand, work responsibilities and family obligations were the main impediments for another 5 participants, who had an average age of 57.3 years. The other 2 participants discontinued due to health-related concerns.

These observations reveal a potential challenge regarding home VF monitoring across a more diverse population. The more senior patients, who are often at higher risk of glaucoma progression and therefore would benefit the most from home monitoring, appear to encounter larger challenges adapting to the technology. Conversely, younger patients, who typically are more familiar with technology and smartphones, tend to have reduced motivation for regular monitoring due to other duties. Therefore, to cope with this situation, a risk-stratified recruitment strategy for long-term home VF monitoring would be important, focusing on patients at higher risk of progression or those with an immediate need for continuous monitoring. However, these technological barriers can be temporary or generational, as familiarity with mobile devices increases over time.

### Cost Analysis

Previous research has shown that the annual average cost to the Canadian health care system in 2001 to treat a glaucoma patient was $508 (in Canadian dollars),^25^ resulting in an estimated total cost of over $220 million per year. Moreover, the cost of glaucoma treatment tends to rise with increasing severity of the disease.^25^, ^26^, ^27^ In addition to treatment cost, the questionnaires used in this study shed light on the considerable time and cost associated with attending clinical visits. On average, participants spent 3.5 hours on transportation to reach the clinical VF testing site, incurring an average cost of $50 per visit per patient. Notably, for some patients (N = 3), the time spent on transportation exceeded 10 hours, with an average cost of over $200. These figures do not even account for the additional expenses related to taking time off from work (N = 7) or having accompanying individuals (N = 3).

In light of these findings, it is apparent that utilizing low-cost home monitoring devices with comparable fidelity and consistency to medical-grade equipment, coupled with frequent testing, can substantially reduce direct costs (such as travel expenses and clinical fees) and indirect costs (such as potentially delayed detection and intervention). However, conducting a comprehensive cost analysis presents significant challenges. In our case, the primary limitation lies in the inability to adequately assess patient outcomes, as a 2-year study does not provide sufficient time to capture long-term effects. Moreover, we lack ethical clearance to conduct comparative analyses between patients adhering to home monitoring protocols and those who do not. Future research should explore these aspects in more depth to fully quantify the cost-effectiveness of home monitoring systems.

### Study Limitations

Our study involved a pool of 25 patients, with 18 actively participating in the long-term home VF test. The small sample size poses challenges in generalizing our findings as well as identifying glaucoma progression over the 2-year duration. Our analyses, which demonstrate improvements with both intertest repeatability and precision in measuring VF progression rates through frequent tests, suggest that a larger and more targeted participant pool could improve the ability to detect significant VF changes with home monitoring. Additionally, our cohort was limited to stable glaucoma patients who were not scheduled for any therapeutic interventions, potentially excluding those at higher risk of progression. Consequently, the progression analysis was performed as a simulation rather than using actual observed data. Another limitation arises from a change in the HFA testing algorithm during the study, transitioning from 24-2 SITA Standard to 24-2C SITA Faster. Although good agreement has been observed between these 2 algorithms,^28^^,^^29^ it remains a limitation in interpreting the results.

In conclusion, this study demonstrates the feasibility of glaucoma patients performing reliable VF tests using the TPP over the long term at home. Visual field results obtained from TPP were found to be in good agreement with results obtained from HFA, suggesting that home testing with TPP holds promise as a viable alternative to clinical VF tests. Technological barriers, time constraints, retention, and compliance were identified as potential challenges with regard to wider adoption of this technology. Participants, however, overwhelmingly expressed a greater preference for conducting VR-based tests at home. While we were not able to demonstrate the detection of VF progression, simulation analyses based on clinical data showed that frequent testing with improved repeatability enhances the sensitivity to detect VF progression. These advantages suggest benefits associated with using TPP for long-term VF home monitoring in glaucoma patients.

## Supplementary Data

Supplementary Method 1

Supplementary Method 2

## References

[1] Stein J.D., Khawaja A.P., Weizer J.S. (2021). Glaucoma in adults—screening, diagnosis, and management: a review. JAMA.

[2] Chauhan B.C., Malik R., Shuba L.M. (2014). Rates of glaucomatous visual field change in a large clinical population. Invest Ophthalmol Vis Sci.

[3] Chauhan B.C., Garway-Heath D.F., Goñi F.J. (2008). Practical recommendations for measuring rates of visual field change in glaucoma. Br J Ophthalmol.

[4] Anderson A.J., Bedggood P.A., Kong Y.X. (2017). Can home monitoring allow earlier detection of rapid visual field progression in glaucoma?. Ophthalmology.

[5] Eizenman M., Shi R.B., Fee T.L. (2018). Visual field testing on a personal smartphone. Invest Ophthalmol Vis Sci.

[6] Ahmed Y., Pereira A., Bowden S. (2022). Multicenter comparison of the Toronto portable perimeter with the Humphrey field analyzer: a pilot study. Ophthalmol Glaucoma.

[7] Matsumoto C., Yamao S., Nomoto H. (2016). Visual field testing with head-mounted perimeter ‘imo. PLoS One.

[8] Stapelfeldt J., Kucur Ş.S., Huber N. (2021). Virtual reality–based and conventional visual field examination comparison in healthy and glaucoma patients. Trans Vis Sci Technol.

[9] McLaughlin D.E., Savatovsky E.J., O'Brien R.C. (2024). Reliability of visual field testing in a telehealth setting using a head-mounted device: a pilot study. J Glaucoma.

[10] Jones P.R. (2020). An open-source static threshold perimetry test using remote eye-tracking (Eyecatcher): description, validation, and preliminary normative data. Trans Vis Sci Technol.

[11] Prea S.M., Kong Y.X., Mehta A. (2018). Six-month longitudinal comparison of a portable tablet perimeter with the Humphrey field analyzer. Am J Ophthalmol.

[12] Jones P.R., Campbell P., Callaghan T. (2021). Glaucoma home monitoring using a tablet-based visual field test (Eyecatcher): an assessment of accuracy and adherence over 6 months. Am J Ophthalmol.

[13] Prea S.M., Kong G.Y., Guymer R.H., Vingrys A.J. (2021). Uptake, persistence, and performance of weekly home monitoring of visual field in a large cohort of patients with glaucoma. Am J Ophthalmol.

[14] Prea S.M., Vingrys A.J., Kong G.Y. (2022). Test reliability and compliance to a twelve-month visual field telemedicine study in glaucoma patients. J Clin Med.

[15] Heijl A., Lindgren G., Olsson J. (1987).

[16] Bengtsson B., Heijl A. (2008). A visual field index for calculation of glaucoma rate of progression. Am J Ophthalmol.

[17] Marín-Franch I., Swanson W.H. (2013). The visualFields package: a tool for analysis and visualization of visual fields. J Vis.

[18] Bland M. (2015).

[19] Raunig D.L., McShane L.M., Pennello G. (2015). Quantitative imaging biomarkers: a review of statistical methods for technical performance assessment. Stat Methods Med Res.

[20] Russell R.A., Garway-Heath D.F., Crabb D.P. (2013). New insights into measurement variability in glaucomatous visual fields from computer modelling. PLoS One.

[21] Li Y., Eizenman M., Shi R.B. (2023). A data-driven model for simulating longitudinal visual field tests in glaucoma. Trans Vis Sci Technol.

[22] Monaghesh E., Hajizadeh A. (2020). The role of telehealth during COVID-19 outbreak: a systematic review based on current evidence. BMC Publ Health.

[23] Aboobakar I.F., Friedman D.S. (2021). Home monitoring for glaucoma: current applications and future directions. Semin Ophthalmol.

[24] Bellsmith K.N., Gale M.J., Yang S. (2022). Validation of home visual acuity tests for telehealth in the COVID-19 era. JAMA Ophthalmol.

[25] Iskedjian M., Walker J., Vicente C. (2003). Cost of glaucoma in Canada: analyses based on visual field and physician's assessment. J Glaucoma.

[26] Traverso C.E., Walt J.G., Kelly S.P. (2005). Direct costs of glaucoma and severity of the disease: a multinational long term study of resource utilisation in Europe. Br J Ophthalmol.

[27] Kobelt-Nguyen G., Gerdtham U.G., Alm A. (1998). Costs of treating primary open-angle glaucoma and ocular hypertension: a retrospective, observational two-year chart review of newly diagnosed patients in Sweden and the United States. J Glaucoma.

[28] Callan T., Yu S., Lee G.C. (2018). Evaluation of the SITA Faster 24-2C visual field test. Invest Ophthalmol Vis Sci.

[29] Lee G.C., Yu S., Callan T. (2019). Diagnostic efficacy of 24-2 and 24-2C SITA Faster global summary indices. Invest Ophthalmol Vis Sci.

