# Supplementary material for: Virtual Reality Portable Perimetry and Home Monitoring of Glaucoma: Retention and Compliance over a 2-year Period
Source: Ophthalmol Sci. 2024 Oct 29;5(2):100639. doi: 10.1016/j.xops.2024.100639 (PMC11634999; doi:10.1016/j.xops.2024.100639)
Supplement: Supplementary Method 1 [file mmc1.pdf]

Supplementary Method 1: The questionnaire of visual field home monitoring study using the Toronto Portable Perimeter

<u>Patient related outcome measure questionnaire to compare subjective patient experience utilizing Toronto Portable Perimeter in comparison to the Humphrey Field Analyzer</u>

1) Which test instructions were easier to understand?
a) TPP
b) HFA
c) No difference

2) Which test did you find easier to perform?
a) TPP
b) HFA
c)No difference

3) Which test produced the least amount of anxiety?
a) TPP
b) HFA
c)No difference

4) If you were to complete multiple visual field tests, which test would you prefer?
a) TPP
b) HFA
c) No preference

5) What is your cost to and from the hospital for each visual field test?
----CAD

6) How much time do you spend travelling to the hospital, and then back home, for a visual field follow-up visit?
----hours

8) If you are currently employed, do you take time off from work to complete an in-hospital visual field test?
a) Yes
b) No

9) If your answer to Q8 is yes, is the work leave…

a) Paid

b) Unpaid

c) N/A

10) Do you require someone to accompany you to the hospital during follow-up visits?

a) Yes

b) No

11) If the answer to the above question is yes, is it a…

a) Family member

b) Caregiver

c) Other

d) N/A

12) If the answer to Q11 is a family member, does he or she have to take time off from work?

a) Yes

b) No

c) N/A

13) If the answer to question no.11 was a caregiver, do you have to pay the caregiver?

a) Yes

b) No

c) N/A

14) If given a choice, would you prefer to perform your visual field test in the hospital or at home?

a) In the hospital

b) At home

c) No difference
