# Supplementary material for: Virtual Reality Portable Perimetry and Home Monitoring of Glaucoma: Retention and Compliance over a 2-year Period
Source: Ophthalmol Sci. 2024 Oct 29;5(2):100639. doi: 10.1016/j.xops.2024.100639 (PMC11634999; doi:10.1016/j.xops.2024.100639)
Supplement: Supplementary Method 2 [file mmc2.pdf]

## Supplementary Method 2: Simulation Analyses for Detecting Visual Field Progression with Home-based Data.

In this study, we conducted a simulation analysis to investigate the expected performance of detecting visual field (VF) progression using data from home-based VF tests with the Toronto Portable Perimeter (TPP). Our simulations were based on earlier methods concerning the simulation of longitudinal VF data.[1,2,3]

To represent statistical features of high-frequency home VF tests, we first derive the empirical noise distribution (test-retest variability) for mean deviations (MD) from VF tests performed with the TPP. Linear regression lines were fitted to the longitudinal MD values for each eye. The true MD was determined from the fitted values, and MD noise calculated by subtracting the true MD from the measured MD at each time point.[3] As such, empirical noise distributions for true MDs (rounded to the nearest decibel) were constructed by pooling noise from different regression lines.[2,3] Next, we determined the empirical testing compliance for home monitoring. The compliance rate for each patient was calculated by dividing the completed tests by 52 (theoretical number of tests over two years). The value was capped at 1 for patients exceeding 52 tests, and the missing rate was defined as the complement of the compliance rate.

Subsequently, longitudinal MD measurements from TPP home tests were simulated using the parameters of simulation length, test frequency, and true progression rate.[4] For each simulated sequence, both the initial MD and the compliance rate values were randomly sampled from the empirical data of the home monitoring study. True MD values at simulated time points were calculated based on the specified true progression rate and sampled initial MD. Noise was added based on the corresponding noise distributions to simulate noisy MD sequence with 100% compliance.[1,2] Finally, to simulate the worsening of compliance over time observed in our study, we estimated time-varying compliance for all patients, constituting a pool of compliance

rate time series (namely compliance pattern). For each simulated MD sequence, a compliance rate time series was randomly selected from the pool. Based on this compliance series, (1 - compliance rate)% of tests were deleted to represent the compliance pattern of home VF testing.

We simulated 10,000 TPP-MD sequences for varying true progression rates (0, –0.5, –1.0, –1.5, and –2.0 dB/year) and testing frequencies (semi-annual and fortnight), respectively. Progress was detected based on the criteria of MD linear regression slope < 0 and P-value < 0.05.[4] The percentage of positive detections at each time point $t$ was determined using data up to year $t$. The detection specificity, sensitivity, and time to detect 80% progression were calculated. The entire process was repeated for the simulation of HFA tests with the noise distributions derived from empirical HFA data.

## Supplementary Method 2 References:


[1] Russell RA, Garway-Heath DF, Crabb DP. New insights into measurement variability in glaucomatous visual fields from computer modelling. PloS one. 2013 Dec 30;8(12):e83595.

[2] Li Y, Eizenman M, Shi RB, Buys YM, Trope GE, Wong W. A Data-Driven Model for Simulating Longitudinal Visual Field Tests in Glaucoma. Translational Vision Science & Technology. 2023 Jun 1;12(6):27-.

[3] Russell RA, Crabb DP, Malik R, Garway-Heath DF. The relationship between variability and sensitivity in large-scale longitudinal visual field data. Investigative Ophthalmology & Visual Science. 2012 Sep 1;53(10):5985-90.

[4] Anderson AJ, Bedggood PA, Kong YX, Martin KR, Vingrys AJ. Can home monitoring allow earlier detection of rapid visual field progression in glaucoma?. Ophthalmology. 2017 Dec 1;124(12):1735-42.
